# Supplementary material for: Hybrid Models and Biological Model Reduction with PyDSTool
Source: PLoS Comput Biol. 2012 Aug 9;8(8):e1002628. doi: 10.1371/journal.pcbi.1002628 (PMC3415397; doi:10.1371/journal.pcbi.1002628)
Supplement: Text S4 — Complete source code for the PyDSTool package (version 0.88.120504). Includes API documentation and help files linking to web pages. This file is identical to the current public release on Sourceforge.net. (ZIP) [file pcbi.1002628.s004.zip › PyDSTool/html/PyDSTool.Toolbox.prep_boxplot-pysrc.html]

xml version="1.0" encoding="ascii"?


PyDSTool.Toolbox.prep\_boxplot


| Home | Trees | Indices | Help | PyDSTool |
| --- | --- | --- | --- | --- |

Package PyDSTool ::
Package Toolbox ::
Module prep\_boxplot

[hide private]

[frames] | no frames]

# Source Code for Module PyDSTool.Toolbox.prep\_boxplot

```
  1  from PyDSTool import remain, loadObjects, array, save_fig, arange, args 
  2  from matplotlib.font_manager import FontProperties 
  3  from matplotlib.pylab import figure, plot, hold, \ 
  4       axes, title, xlabel, ylabel, draw, \ 
  5       boxplot, text 
  6  from scipy import mean 
  7   
  8  ##symbol_map = {'isomap': { 
  9  ##                    'K': {10: 'k<', 50: 'k>', 100: 'k^', 500: 'kv'}, 
 10  ##                    'eps': {20: 'w<', 50: 'w>', 100: 'w^'} 
 11  ##                        }, 
 12  ##              'pca': { 
 13  ##                  'knee': {1: 'ws', 2: 'ks'}, 
 14  ##                  'var': {80: 'wo', 90: 'ko'} 
 15  ##                     } 
 16  ##              } 
 17   
 18  symbol_map = {'isomap': { 
 19                      'K': {10: ('ws', 'E'), 50: ('ws', 'F'), 
 20                            100: ('ws', 'G'), 500: ('ws', 'H')}, 
 21                      'eps': {20: ('ws', 'I'), 50: ('ws', 'J'), 
 22                              100: ('ws', 'K')} 
 23                          }, 
 24                'pca': { 
 25                    'knee': {1: ('wo', 'A'), 2: ('wo', 'B')}, 
 26                    'var': {80: ('wo', 'C'), 90: ('wo', 'D')} 
 27                       } 
 28                } 
 29   
 30   
 31  # For use with PD-E analysis 


32 -def prep_boxplots(data, xlabel_str, figname='', fignum=1, do_legend=1, 
 33                    means=1, xlegoff=0, ylegstep=1, ylegoff=0, spacing=None):


34      spacing_actual = { 
 35          'width': 0.1, 
 36          'wgapfac': 0.75, 
 37          'markersize': 12, 
 38          'off_fac': 0.7, 
 39          'x_step': 0.9,   # 9 * width 
 40          'x_off': 0, 
 41          'box_to_marker': 1.1, 
 42          'notch_size': 0.2} 
 43      if spacing is not None: 
 44          spacing_actual.update(spacing) 
 45      width = spacing_actual['width'] 
 46      wgapfac = spacing_actual['wgapfac'] 
 47      markersize = spacing_actual['markersize'] 
 48      off_fac = spacing_actual['off_fac'] 
 49      x_step = spacing_actual['x_step'] 
 50      x_off = spacing_actual['x_off'] 
 51      box_to_marker = spacing_actual['box_to_marker'] 
 52      notch_size = spacing_actual['notch_size'] 
 53   
 54      n = len(data) 
 55      x_min = -width*3.8 #3.75 
 56      x_max = (n-1)*x_step+width*4.5 #3.75 
 57      if n > 1: 
 58          halfticks = arange(1,n)*x_step-x_step/2 
 59      figure(fignum) 
 60      # work out ordering of data from 'pos' key 
 61      order = {} 
 62      # `pos` position runs from 1 to n, `ns` runs from 0 to n-1 
 63      ns = [] 
 64      for k, v in data.iteritems(): 
 65          order[v['pos']] = k 
 66          ns.append(v['pos']-1) 
 67      ns.sort() 
 68      assert ns == range(n) 
 69      maxD = 0 
 70      max_dimval_markers = 0 
 71      labels = [] 
 72      for pos in range(n): 
 73          name = order[pos+1] 
 74          pde_name = 'PD_E-'+name 
 75          if 'known_dim' in data[name]: 
 76              if n == 1: 
 77                  kdx1 = x_min 
 78                  kdx2 = x_max 
 79              else: 
 80                  if pos == 0: 
 81                      kdx1 = x_min 
 82                      kdx2 = halfticks[0] 
 83                  elif pos == n-1: 
 84                      kdx1 = halfticks[n-2] 
 85                      kdx2 = x_max 
 86                  else: 
 87                      kdx1 = halfticks[pos-1] 
 88                      kdx2 = halfticks[pos] 
 89              plot([[kdx1], [kdx2]], 
 90                   [data[name]['known_dim'],data[name]['known_dim']], 
 91                   'k', linewidth=1, zorder=0) 
 92          slope_data = loadObjects(pde_name)[2] 
 93          ds_mins = array(slope_data[:,0])#,shape=(len(slope_data),1)) 
 94          ds_mins.shape=(len(slope_data),1) 
 95          ds_maxs = array(slope_data[:,1])#,shape=(len(slope_data),1)) 
 96          ds_maxs.shape=(len(slope_data),1) 
 97          max_ds = max([max(ds_mins[:,0]),max(ds_maxs[:,0])]) 
 98          if max_ds > maxD: 
 99              maxD = max_ds 
100          # limits args are ineffective here 
101          boxplot(ds_mins,positions=[pos*x_step-width*wgapfac+x_off],whis=100, 
102                  means=means,monochrome=True,notch=2,notchsize=notch_size, 
103                  limits=(),widths=width,fill=1) 
104          boxplot(ds_maxs,positions=[pos*x_step+width*wgapfac+x_off],whis=100, 
105                  means=means,monochrome=True,notch=2,notchsize=notch_size, 
106                  limits=(),widths=width,fill=1) 
107          if pos == 0: 
108              fa = figure(fignum).axes[0] 
109              fa.hold(True) 
110          if means: 
111              ds_all_mean = (mean(ds_mins[:,0])+mean(ds_maxs[:,0]))/2 
112              plot([pos*x_step+x_off], [ds_all_mean], 'k^', 
113                   markersize=markersize-2) 
114          pca_x = pos*x_step-width*(wgapfac+box_to_marker)+x_off 
115          isomap_x = pos*x_step+width*(wgapfac+box_to_marker)+x_off 
116          pca_ds = {} 
117          isomap_ds = {} 
118          try: 
119              pca_data = data[name]['pca'] 
120          except KeyError: 
121              pca_data = [] 
122          pca_ds, max_dimval_pca, pca_used = plot_markers(pca_data, 
123                                            pca_x, 'PCA', 
124                                            symbol_map['pca'], -1, 
125                                            width, off_fac, markersize) 
126          if max_dimval_pca > maxD: 
127              maxD = max_dimval_pca 
128          if max_dimval_pca > max_dimval_markers: 
129              max_dimval_markers = max_dimval_pca 
130          try: 
131              isomap_data = data[name]['isomap'] 
132          except KeyError: 
133              isomap_data = [] 
134          isomap_ds, max_dimval_iso, isomap_used = plot_markers(isomap_data, 
135                                               isomap_x, 'Isomap', 
136                                               symbol_map['isomap'], 1, 
137                                               width, off_fac, markersize) 
138          if max_dimval_iso > maxD: 
139              maxD = max_dimval_iso 
140          if max_dimval_iso > max_dimval_markers: 
141              max_dimval_markers = max_dimval_iso 
142          labels.append(data[name]['label']) 
143      ## legend 
144      if do_legend: 
145          font = FontProperties() 
146          font.set_family('sans-serif') 
147          font.set_size(11) 
148          x_legend = x_min + 3*width/4 + xlegoff 
149          y_legend = maxD+ylegoff 
150          # pca legend 
151          for k, s in pca_used: 
152              plot_markers([(k,s,y_legend)], x_legend, 'Legend', symbol_map['pca'], 
153                       1, width, off_fac, markersize) 
154              if k == 'var': 
155                  legstr = "%s=%d%%"%(k,s) 
156              else: 
157                  legstr = "%s=%d"%(k,s) 
158              text(x_legend+3*width/4, y_legend-width*2., legstr, 
159                   fontproperties=font) 
160              y_legend -= ylegstep 
161          # isomap legend 
162          isomap_leg_data = [] 
163          for k, s in isomap_used: 
164              if y_legend-width*2. <= max_dimval_markers + 2: 
165                  y_legend = maxD+ylegoff 
166                  x_legend += x_step #-width*.75 
167              plot_markers([(k,s,y_legend)], x_legend, 'Legend', symbol_map['isomap'], 
168                        1, width, off_fac, markersize) 
169  ##        if k == 'eps': 
170  ##            kstr = '\\epsilon' 
171  ##        else: 
172  ##            kstr = k  
173              text(x_legend+3*width/4, y_legend-width*2., "%s=%d"%(k,s), 
174                   fontproperties=font) 
175              y_legend -= ylegstep 
176      ## tidy up axes, etc. 
177      fa.set_xticks(arange(n)*x_step) 
178      if n>1: 
179          for h in range(n-1): 
180              plot([halfticks[h], halfticks[h]], [0,maxD+1+ylegoff], 'k:') 
181      fa.set_xticklabels(labels) 
182      fa.set_position([0.07, 0.11, 0.9, 0.85]) 
183      fa.set_xlim(x_min,x_max) 
184      fa.set_ylim(0,maxD+1+ylegoff) 
185      if xlabel_str != '': 
186          xlabel(r'$\rm{'+xlabel_str+r'}$',args(fontsize=20,fontname='Times')) 
187      ylabel(r'$\rm{Dimension}$',args(fontsize=20,fontname='Times')) 
188      draw() 
189      if figname != '': 
190          save_fig(fignum, figname)

191   
192   


193 -def plot_markers(data, x_base, name, map, xoff_sgn, width, off_fac, 
194                   markersize):


195      maxD = 0 
196      ds = {} 
197      used = [] 
198      font = FontProperties() 
199      font.set_family('sans-serif') 
200      font.set_size(10) 
201      for (kind, subkind, dimval) in data: 
202          try: 
203              symb, lab = map[kind][subkind] 
204          except KeyError: 
205              raise KeyError("Invalid key for %s symbols"%name) 
206          used.append((kind, subkind)) 
207          try: 
208              ds[dimval] += 1 
209              x_off = xoff_sgn*width*off_fac*(ds[dimval]-1) 
210          except KeyError: 
211              ds[dimval] = 1 
212              x_off = 0 
213          plot([x_base+x_off], [dimval], symb, markersize=markersize) 
214          # hack tweaks 
215      ##            if lab=='C': 
216      ##                x_off -= width/15 
217          if lab=='A': 
218              x_off += width/30 
219          text(x_base+x_off-width*.15, dimval-width*2., lab, 
220               fontproperties=font) 
221          if dimval > maxD: 
222              maxD = dimval 
223      return ds, maxD, used

224
```

  


| Home | Trees | Indices | Help | PyDSTool |
| --- | --- | --- | --- | --- |

|  |  |
| --- | --- |
| Generated by Epydoc 3.0.1 on Fri May 4 15:24:18 2012 | http://epydoc.sourceforge.net |
